# Fabrication of ZnS-Bi-TiO_2_ Composites and Investigation of Their Sunlight Photocatalytic Performance

**DOI:** 10.1155/2014/503895

**Published:** 2014-02-11

**Authors:** Xuewei Dong, Fan Zhang, Chuan Rong, Hongchao Ma

**Affiliations:** ^1^School of Environmental and Chemical Engineering, Dalian Jiaotong University, Dalian 116028, China; ^2^School of Light Industry and Chemical Engineering, Dalian Polytechnic University, Dalian 116034, China

## Abstract

The ZnS-Bi-TiO_2_ composites were prepared by the sol-gel method and were characterized by X-ray photoelectron spectroscopy (XPS), transmission electron microscopy (TEM), X-ray diffraction (XRD) and UV-visible diffuse reflectance spectroscopy (UV-Vis DRS). It is found that the doped Bi as Bi^4+^/Bi^3+^ species existed in composites, and the introducing of ZnS enhanced further the light absorption ability of TiO_2_ in visible region and reduced the recombination of photogenerated electrons and holes. As compared to pure TiO_2_, the ZnS-Bi-TiO_2_ exhibited enhanced photodegradation efficiency under xenon lamp irradiation, and the kinetic constant of methyl orange removal with ZnS-Bi-Ti-0.005 (0.0141 min^−1^) was 3.9 times greater than that of pure TiO_2_ (0.0029 min^−1^), which could be attributed to the existence of Bi^4+^/Bi^3+^ species, the ZnS/TiO_2_ heterostructure.

## 1. Introduction

Oxide semiconductors depended on their excellent performance in photocatalytic realm and has drawn scientific interests for ongoing research. Among various oxide semiconductor photocatalysts, TiO_2_ has been widely considered as the most promising photocatalyst owing to its high photocatalytic activity, good chemical and biological stability, nontoxicity, low cost, and so forth [1–4]. However, TiO_2_ could only be excited under UV irradiation due to its large energy band gap of 3.2 eV, which implied that only about 5% of solar energy can be utilized by TiO_2_ [5–8]. In recent years, great interests have been focused on the development of photocatalysts with visible light response.

The presence of metal ion dopants in the TiO_2_ crystalline matrix significantly influences its photocatalytic properties. It has been reported that doping TiO_2_ with metal may extend its light absorption further into the visible region [9–15]. When TiO_2_ was doped by metal ion, new impurity levels could be introduced between the conduction and valence band of TiO_2_, and thus narrowing the band gap of TiO_2_ [16]. Xu et al. [17] synthesized the Bi-doped TiO_2_ by an electrospinning method, and the Bi-doped TiO_2_ exhibited higher activities than sole TiO_2_ in the degradation of rhodamine B. Coupled TiO_2_ with other semiconductors also has been confirmed as an effective approach to enhance the visible light response. When both semiconductors are illuminated, electrons accumulate at the low-lying conduction band of one semiconductor while holes accumulate at the valence band of the other compound. These processes of charge separation are very fast and the efficiency of reduction or oxidation of the adsorbed organics remarkably increases. Yu et al. [18] successfully synthesized the ZnS/TiO_2_ via microemulsion-mediated solvothermal method, and the ZnS/TiO_2_ exhibited enhanced visible light photocatalytic activity for the aqueous parathion-methyl degradation.

In this study, we integrated the above two methods and successfully synthesized the ZnS-Bi-TiO_2_ photocatalyst by sol-gel method. Methyl orange (MO), which is a common pollutant in the industry effluents, was chosen to test the photocatalytic ability of ZnS-Bi-TiO_2_.

## 2. Experiment

### 2.1. Preparation of ZnS-Bi-TiO_2_

#### 2.1.1. Preparation of ZnS

All chemicals were used as received without further purification. The equal molar quantities of ZnCl_2_ and Na_2_S·9H_2_O were put into the agate mortar and fully grinded, then the yellow solid was obtained. After the solid was washed, dried, and groud, the ZnS nanoparticle was gained.

#### 2.1.2. Preparation of ZnS-Bi-TiO_2_

40 mL absolute ethyl alcohol and 0.2 mL HNO_3_ were added into 0.05 mol Tetra-n-butyl titanate (TnBT), then a certain amount of ZnS was put into the mixture, and then solution A was obtained. Solution B consisted of 20 mL absolute ethyl alcohol, 7 mL deionized water, 7.5 mL glacial acetic acid, and a constant amount of Bi(NO_3_)_3_·5H_2_O (Bi/Ti = 0.006). After solution A was ultrasonic treated for 15 min and stirred for 30 min, solution B was slowly dripped into solution A. The resulting colloid was aged at room temperature for 48 h; dried at 100°C for 7 h, and then calcined in tubular furnace at 400°C with protection of nitrogen for 5 h. Finally, the samples were obtained and named as ZnS-Bi-TiO_2_-*x*, where *x* denotes the molar ratio of ZnS to Ti.

### 2.2. Characterization

The composition of the samples was identified by X-ray photoelectron spectroscopy (XPS, ESCALAB250, Thermo VG) with Al K*α* radiation. The microstructure of the samples was characterized by transmission electron microscopy (TEM, JEM-2000EX, JEOL). The crystallinity of the samples was identified by X-ray diffraction analysis (XRD, XRD-6100, SHIMADZU) using graphite-monochromatized Cu K*α* radiation at 40 kV, 30 mA. Light absorption property was evaluated by UV-Vis diffuse reflectance spectroscopy (UV-Vis DRS, CARY 100&300, VARIAN). The PL spectra of photocatalysts were measured using a fluorescence spectrophotometer (PE-LS55, USA) equipped with a xenon lamp at an excitation wavelength of 325 nm.

### 2.3. Photocatalytic Experiment

The photocatalytic activity of ZnS-Bi-TiO_2_ was evaluated by degradation of MO. The reaction was carried out in the multifunctional photochemical reaction instrument equipped with a 350 W xenon lamp. In each experiment, MO solution (*C*_0_ = 20 mg/L, 200 mL) was added into the instrument, and 0.4 g of ZnS-Bi-TiO_2_ was suspended in the solution. At every 20 min, a certain volume of suspension was sampled and centrifuged to remove the particles. The concentration of MO was determined by UV-Vis spectrophotometer at 464 nm.

The stability of ZnS-Bi^3+^-TiO_2_ was tested by repeating the same experiment for four times. Once each run of photodegradation experiment was finished, the used ZnS-Bi-TiO_2_ was centrifuged and washed with ethanol and deionized water for certain times, and then dried before reuse.

## 3. Results and Discussion

**Figure 1:**
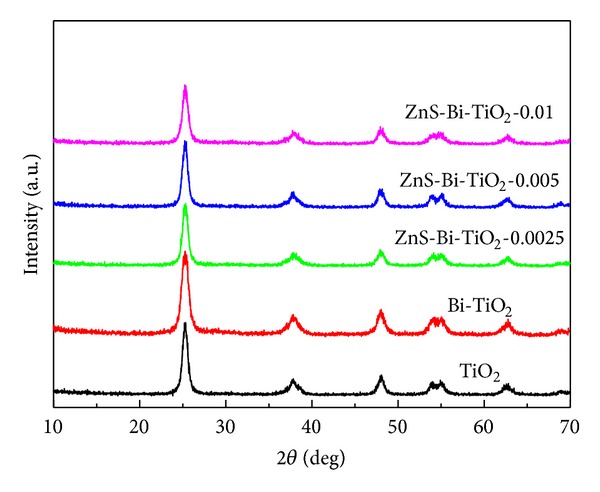
XRD patterns of TiO_2_, Bi-TiO_2_, and ZnS-Bi-TiO_2_-*x*.

**Figure 2:**
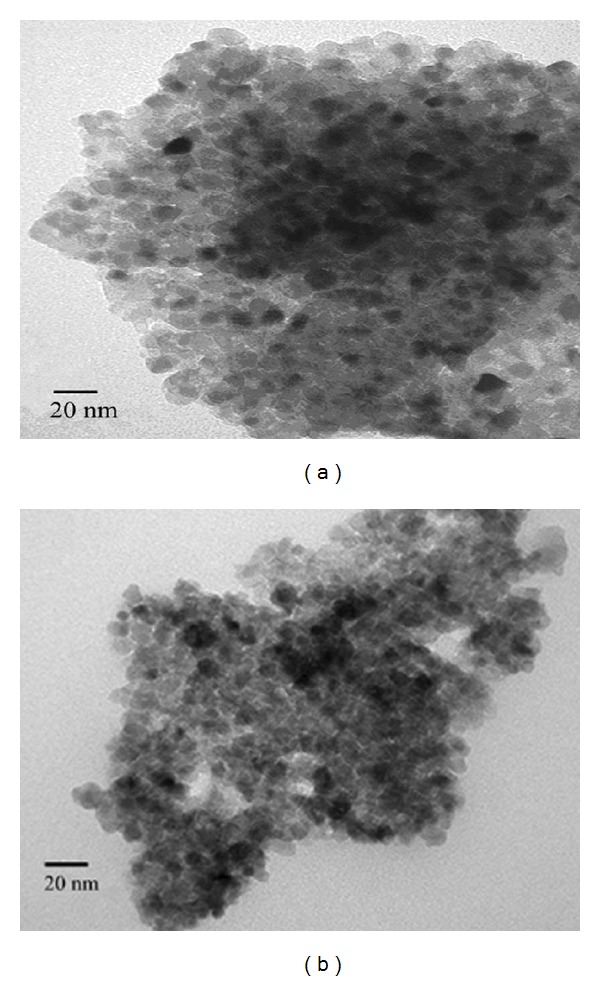
TEM micrograph of (a) TiO_2_ and (b) ZnS-Bi-TiO_2_-0.005.

As shown in Figure 1, all diffraction peaks can be attributed to the TiO_2_ with anatase crystal structure. No peaks corresponding to bismuth oxide and ZnS phases were detected in the XRD patterns of the ZnS-Bi-TiO_2_-*x*, which might be due to low amount of Bi^3+^ and ZnS. The mean crystal size of ZnS-Bi-TiO_2_-*x* samples shown in Table 1 was calculated by the Scherrer equation (the {101} peak of samples was taken into account for the Scherrer calculation). The crystal size of the Bi-TiO_2_ was smaller than that of TiO_2_, which suggested that doping TiO_2_ with Bi^3+^ could suppress the crystal growth of TiO_2_ during the annealing process [1]. In order to investigate the nanocrystal morphology and structure of the catalyst, the TEM observation for TiO_2_ and ZnS-Bi-TiO_2_-0.005 samples was carried out. It can be seen from Figures 2(a) and 2(b) that the comodification of Bi and ZnS did not change the morphology of TiO_2_, and the size of TiO_2_ and ZnS-Bi-TiO_2_-0.005 particles was about 10 nm (which was similar to the average particle size obtained by XRD analysis).

**Figure 3:**
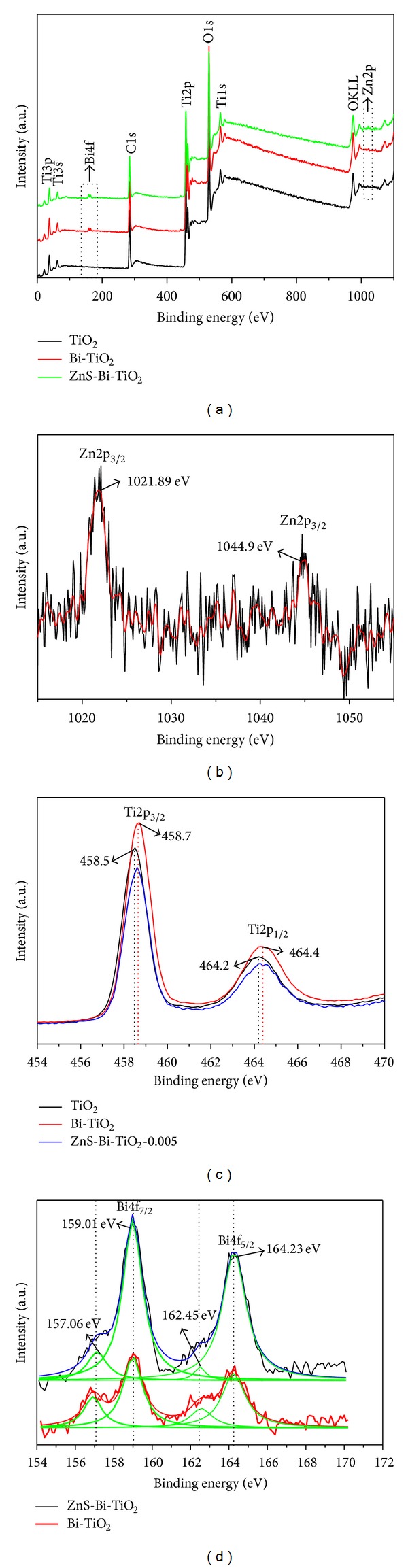
XPS spectra of (a) XPS survey spectra of samples, (b) XPS spectrum of Zn2p, (c) XPS spectrum of Ti2p, and (d) XPS spectrum of Bi4f.

Investigation of the surface chemical compositions and their electronic states of the samples were carried out by XPS. The survey spectra of TiO_2_, Bi-TiO_2_ and ZnS-Bi-TiO_2_-0.005 samples are shown in Figure 3(a). As shown in Figure 3(a), a detectable Bi4f peak could be observed in the Bi-TiO_2_ and ZnS-Bi-TiO_2_-0.005 samples compared to that of pure TiO_2_, which confirmed the successful incorporation of Bi atoms into the composites. Furthermore, the detectable high-resolution peak of Zn2p was observed for ZnS-Bi-TiO_2_-0.005 sample in Figure 3(b). The Zn2p_3/2_ and 2p_1/2_ core levels centered at 1021.89 and 1044.9 eV, which was completely matched with the binding energy of Zn2p in ZnS reported by Brion [19]. It suggested that the ZnS was present mainly as separate phases in ZnS-Bi-TiO_2_-0.005 composite. Although the above XPS results confirmed the existence of Zn element in the ZnS-Bi-TiO_2_-0.005 sample, but it is difficult to identify the S2p peak in ZnS-Bi-TiO_2_-0.005 sample because the binding energies of S2p and Bi4f are very close and due to the low content and sensitivity factor of S element. Nevertheless, the peak of Ti2p centered at 458.7 eV for Bi-TiO_2_ and ZnS-Bi-TiO_2_-0.005 shows a positive shift of approximately 0.2 eV as compared to those of pure TiO_2_ (see Figure 3(c)). This might be due to the fact that Ti atom was probably substituted by Bi atom and the chemical environmental surrounding of Ti may be Ti-O-Bi.

The high-resolution XPS spectrum of Bi in the 4f region for Bi-TiO_2_ and ZnS-Bi-TiO_2_-0.005 samples is displayed in Figure 3(d). There are four peaks centered at 164.23, 162.45, 159.01, and 157.06 eV in the 4f region of Bi. The peaks of 162.45 and 157.06 eV could be attributed to the Bi^3+^ in Bi_2_O_3_, which is in good agreement with other studies [20]. The 164.23 and 159.01 eV peaks at high binding energy could be assigned to the Bi^4+^ doped into the TiO_2_ lattice (the doped Bi is oxidized to Bi^4+^ due to strong interaction with TiO_2_) [21–23]. The presence of Bi^4+^/Bi^3+^ species in the catalyst is favorable to trap electrons, and improves the separation of the electron-hole pairs in the photocatalytic process.

**Figure 4:**
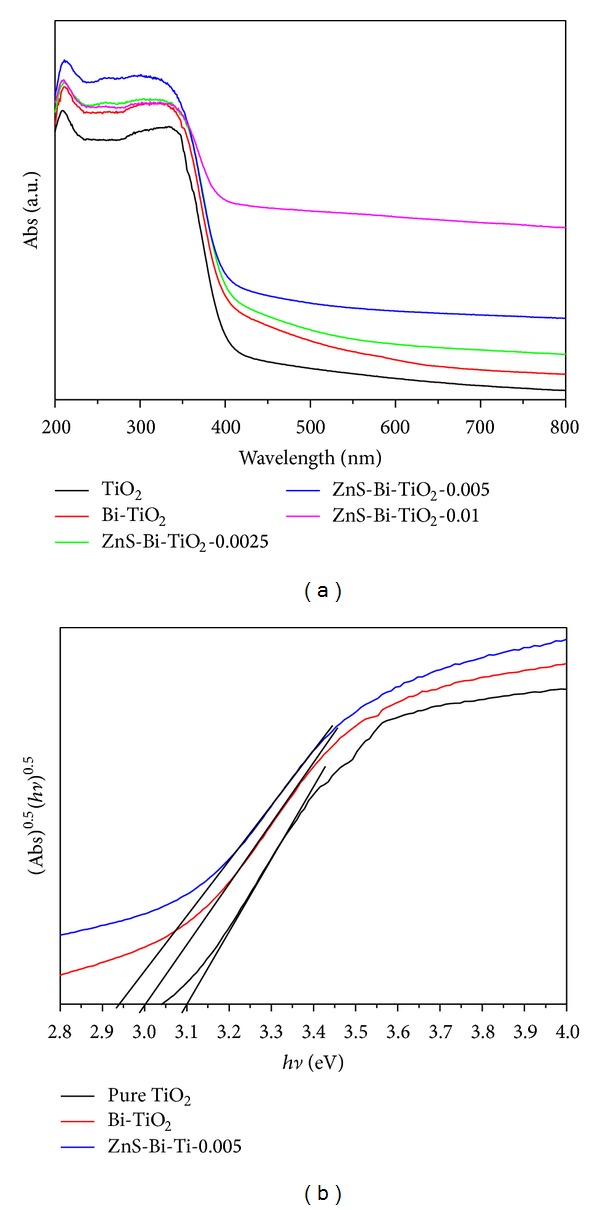
(a) UV-Vis spectra of the TiO_2_, Bi-TiO_2_, and ZnS-Bi-Ti-*x* and (b) the calculation of energy band gap of the TiO_2_, Bi-TiO_2_, and ZnS-Bi-Ti-0.005.

Figure 4(a) shows the UV-Vis absorption spectra of the pure TiO_2_, Bi-TiO_2_, and ZnS-Bi-TiO_2_ with various ZnS content. Compared with the pure TiO_2_, the Bi-TiO_2_ samples showed remarkable absorption in the visible light region and red shift of absorption edge. This wide visible light response of Bi-doped TiO_2_ could be attributed to the formation of surface defect centers, which are associated with existence of oxygen vacancies created by the doping process [24, 25]. The red shift of absorption edge corresponded to the band gap narrowing of TiO_2_ (see Figure 4(b)), which may be ascribed to the introduction of new impurity levels between the conduction and valence band of TiO_2_ by the doping of Bi. After introducing ZnS, the absorption in visible light region is increased and the absorption edge of TiO_2_ was further shifted to the visible light region with the increase of ZnS content. This may be attributed to the sulphur from ZnS is able to dope into the surfaces TiO2 particles [26] and decreases the value of band-gap energy. The enhancement of optical absorption intensity in the visible region for ZnS-Bi-TiO_2_ samples implies that the ZnS-Bi-TiO_2_ composites have better photocatalytic activities than those of pure TiO_2_ and Bi-TiO_2_ under visible light irradiation.

**Figure 5:**
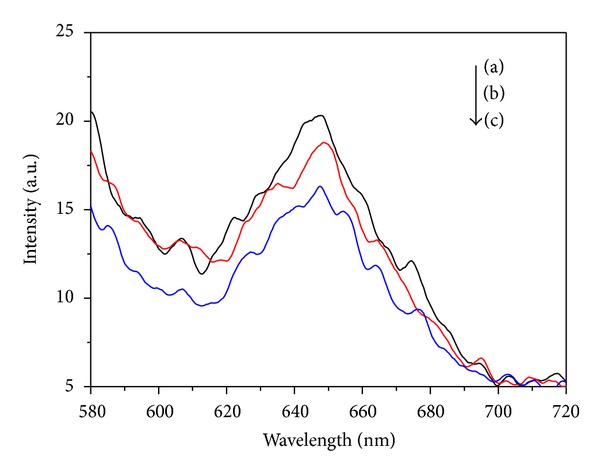
Room temperature photoluminescence of (a) TiO_2_, (b) Bi-TiO_2_, and (c) ZnS-Bi-TiO_2_-0.005.

PL spectra can provide information about the features of excited states and related defects based on the electronic structure and optical characteristics [27].  Figure 5 shows room temperature photoluminescence spectra for pure TiO_2_, Bi-TiO_2_, and ZnS-Bi-TiO_2_-0.005. As shown in Figure 5, the PL intensity decreased with successive introducing of Bi and ZnS. The PL spectra indicate that the recombination rate of photogenerated charge carriers is lower on the surface of the Bi-TiO_2_ and ZnS-Bi-TiO_2_-0.005 samples than pure TiO_2_. The lower PL spectra intensity for composites may be contributed to the doping of Bi and ZnS/TiO_2_ heterostructures in the composites. The Bi impurity energy level as a separation center can avoid volume recombination of photoinduced cariers. Moreover, the ZnS/TiO_2_ heterostructures improved the interfacial charge transfer rate and inhibited the recombination of photoinduced electron-hole pairs. The slower recombination of the photogenerated charges is advantageous for photocatalysis.

**Figure 6:**
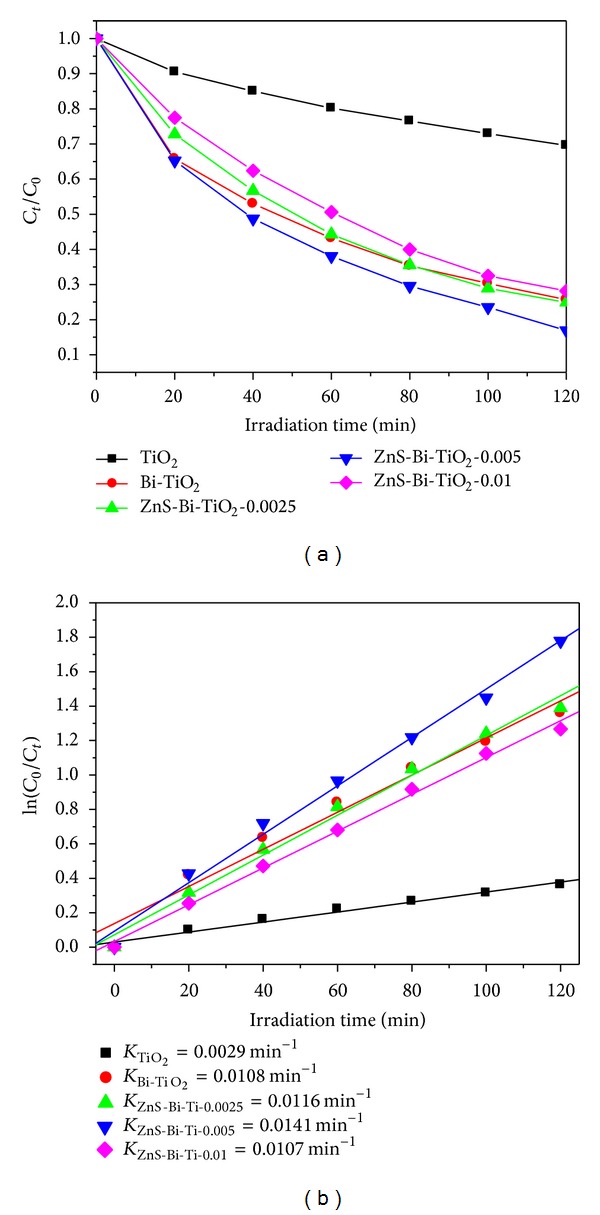
MO concentration (a) and ln⁡(*C*_0_/*C*_*t*_) (b) versus time in photocatalytic process.

The photocatalytic activity of ZnS-Bi-TiO_2_ was evaluated by photocatalytic degradation of MO under simulated sunlight illumination. As shown in Figure 6(a), the Bi-TiO_2_ and ZnS-Bi-TiO_2_-*x* samples exhibited higher photocatalytic activity compared with that of the TiO_2_. It was found that the photocatalytic elimination of MO with TiO_2_, Bi-TiO_2_, and ZnS-Bi-TiO_2_ followed the pseudo-first-order kinetics by formula (1). Consider

(1) $$\begin{array}{c} \ln\left(\frac{C_{t}}{C_{0}}\right)=-kt. \end{array}$$

**Figure 7:**
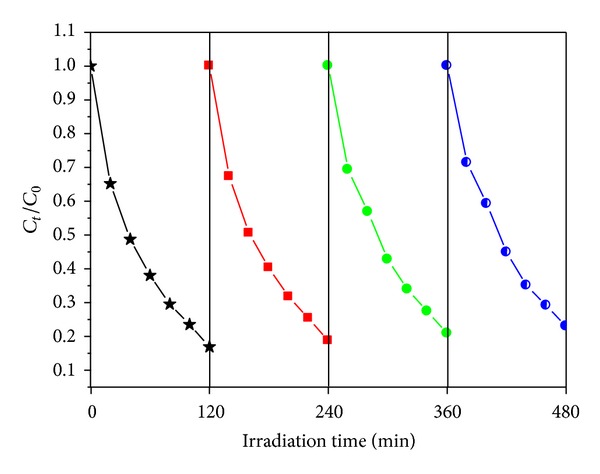
Recycling runs in the photocatalytic degradation of MO.

As shown in Figure 6(b), the *k* value (*k* is kinetic constant) of the Bi-TiO_2_ and ZnS-Bi-TiO_2_-*x* samples was larger than that of TiO_2_. The enhanced photocatalytic performance of the Bi-TiO_2_ and ZnS-Bi-TiO_2_-*x* samples could be attributed to that the Bi doping and heterostructure of ZnS/TiO_2_ could enhance light absorption and separation efficiency of photo-induced electron-hole pairs. It is noteworthy that the ZnS-Bi-TiO_2_-0.005 showed the best MO degradation rate, and *k* value of ZnS-Bi-TiO_2_-0.005 (0.0141 min^−1^) was 4.9 times as great as that of TiO_2_ (0.0029 min^−1^). However, too much loading of ZnS shows adverse effects because some ZnS sites may act as charge recombination centers. Stability is one of the most important performances of photocatalysts, which could make photocatalysts to be reused. In order to evaluate the stability of photocatalysts, ZnS-Bi-TiO_2_-0.005, as a representative sample, was chosen for four recycles of photodegradation of MO. As presented in Figure 7, the photocatalytic activity exhibited no significant decrease after four recycles. Clearly, the photocatalytic activity of the ZnS-Bi-Ti-0.005 was quite stable.

## 4. Conclusion

ZnS-Bi-TiO_2_ photocatalyst with visible light response was fabricated by a facile sol-gel method and its photocatalytic performance was tested by MO degradation under xenon lamp irradiation. Compared to TiO_2_, the remarkable enhancement of photocatalytic capability was achieved, which was attributed to the doped Bi^3+^ and coupled ZnS that improved the ability to visible light absorption by TiO_2_. Furthermore, no significant decrease of activity was observed after four cycles for photodegradation of MO. Considering the photocatalytic ability under sunlight and the stability of ZnS-Bi-TiO_2_, it is believed that ZnS-Bi-TiO_2_ photocatalyst may have potential application in the field of water pollution control.

## Figures and Tables

**Table 1:** Crystallite size (nm) of TiO_2_, Bi-TiO_2_, and ZnS-Bi-TiO_2_-*x*.

Annealing temperature	Samples				
	TiO_2_	Bi-TiO_2_	ZnS-Bi-TiO_2_-0.0025	ZnS-Bi-TiO_2_-0.005	ZnS-Bi-TiO_2_-0.01
400°C	10.7	9.3	10.4	10.3	10.0
